# miR-26a inhibits atherosclerosis progression by targeting TRPC3

**DOI:** 10.1186/s13578-018-0203-9

**Published:** 2018-01-19

**Authors:** Min Feng, Daqian Xu, Lirui Wang

**Affiliations:** Department of Intensive Care Unit, The First Affiliated Hospital of Zhengzhou University, No. 1 of Jian She East Road, Zhengzhou, 450052 China

**Keywords:** miR-26a, TRPC3, ApoE^−/−^, Endothelial cells, ox-LDL, Atherosclerosis

## Abstract

**Background:**

Atherosclerosis, a chronic multi-factorial vascular disease, has become a predominant cause of a variety of cardiovascular disorders. miR-26a was previously reported to be involved in atherosclerosis progression. However, the underlying mechanism of miR-26a in atherosclerosis remains to be further explained.

**Methods:**

High-fat diet (HFD)-fed apolipoprotein E (apoE)^−/−^ mice and oxidized low-density lipoprotein (ox-LDL)-stimulated human aortic endothelial cells (HAECs) were established as in vivo and in vitro models of atherosclerosis. RT-qPCR and western blot analysis were performed to measure the expression of miR-26a and transient receptor potential canonical 3 (TRPC3), respectively. Binding between miR-26a and TRPC3 was predicted with bioinformatics software and verified using a dual luciferase reporter assay. The effects of miR-26a on the lipid accumulation, atherosclerotic lesion, and inflammatory response in HFD-fed apoE^−/−^ mice were investigated by a colorimetric enzymatic assay system, hematoxylin–eosin and oil-Red-O staining, and ELISA, respectively. Additionally, the effects of miR-26a or combined with TRPC3 on cell viability, apoptosis and the nuclear factor-kappa B (NF-κB) pathway in ox-LDL-stimulated HAECs were evaluated by MTT assay, TUNEL assay, and western blot, respectively.

**Results:**

miR-26a was downregulated in HFD-fed apoE^−/−^ mice and ox-LDL-stimulated HAECs. miR-26a overexpression inhibited the pathogenesis of atherosclerosis by attenuating hyperlipidemia, atherosclerotic lesion and suppressing inflammatory response in HFD-fed apoE^−/−^ mice. Moreover, miR-26a overexpression suppressed inflammatory response and the NF-κB pathway, promoted cell viability and inhibited apoptosis in ox-LDL-stimulated HAECs. Additionally, TRPC3 was demonstrated to be a direct target of miR-26a. Enforced expression of TRPC3 reversed the effects of miR-26a on cell viability, apoptosis, and the NF-κB pathway in ox-LDL-treated HAECs.

**Conclusions:**

miR-26a alleviated the development of atherosclerosis by regulating TRPC3, providing a potential target for atherosclerosis treatment.

## Background

Atherosclerosis, a chronic multi-factorial vascular disease, has become a predominant cause of a variety of cardiovascular disorders including heart attacks, ischemic stroke, and peripheral vascular disease, accounting for the significant morbidity and morbidity in aged people in the world [1]. In the early stage of atherosclerosis, endothelial dysfunction results in accumulation of abundant lipids in arterial wall and formation of atherosclerotic plaque [2, 3]. It is commonly thought that endothelial cell (EC) apoptosis, a critical cellular event involved in the pathogenesis of atherogenesis, contributes to endothelial dysfunction and destabilization of atherosclerotic plaques and thrombosis [4, 5]. Therefore, preventing EC apoptosis has received considerable attention as a promising novel therapeutic option against atherosclerosis. Increasing evidence has indicated that among the multi-factorial causes of atherosclerosis, chronic lipid-induced inflammation is crucial for the initiation and progression of atherosclerosis [6]. Thus, elucidating the underlying mechanism involved in the pathogenesis of atherosclerosis is of great significance to develop novel therapeutic strategies for atherosclerosis.

MicroRNAs (miRNAs) belong to a class of small, highly conserved noncoding RNA molecules with about 22 nucleotides in length. miRNAs negatively regulate gene expression at a post-transcriptional level and play a crucial role in pathophysiological processes through complementary binding to the 3′ untranslated region (UTR) of their targets, eventually leading to translational repression or mRNA degradation [7]. Accumulating evidence has indicated the importance of miRNAs as contributing risk factors in the pathogenesis of atherosclerosis by affecting the proliferation, migration, and apoptosis of vascular cells [8, 9]. miR-26a is a highly conserved miRNA that plays essential roles in tumorigenesis and growth, functioning as either a tumor suppressor or oncogene [10]. Dysregulation of miR-26a was frequently observed in cardiovascular diseases such as cardiac hypertrophy [11], atrial fibrillation [12] and myocardial ischemia [13]. Interestingly, it was previously reported that miR-26a expression was markedly downregulated in apolipoprotein E-deficient (apoE^−/−^) mice fed with a high-fat diet (HFD) and forced expression of miR-26a inhibited endothelial apoptosis by targeting transient receptor potential canonical 6 (TRPC6) [14]. However, the detailed role of miR-26a and underlying mechanism in the pathogenesis of atherosclerosis remains to be defined.

TRPC proteins belong to the transient receptor potential canonical (TRPC) superfamily of channel forming proteins and can be grouped into four subfamilies based on their structure and functional similarities: TRPC1, TRPC2, TRPC3/6/7 and TRPC4/5 [15]. TRPC3, a member of TRPC family of Ca^2+^-permeable channels, is an obligatory component in endothelial inflammatory signaling associated to monocyte recruitment, as well as survival mechanism in human and murine macrophages [16]. TRPC3 was also reported to be implicated in lesion development of atherogenesis [17]. Since TRPC6 was previously identified as a target of miR-26a in atherosclerosis development [14], we hypothesized whether miR-26a could also directly target TRPC3 to regulate the pathogenesis of atherogenesis.

In the present study, we aimed to explore the exact role of miR-26a and underlying mechanism in the pathogenesis of atherogenesis in atherosclerotic apolipoprotein E-deficient (apoE^−/−^) mouse model and oxidized low-density lipoprotein (ox-LDL)-treated human aortic endothelial cells (HAECs).

## Methods

### Animal model of atherosclerosis

The animal protocols were approved by the Institutional Animal Care and Use Committee of the First Affiliated Hospital of Zhengzhou University (Zhengzhou, China) and performed according to national legislation and institutional guidelines. Five-week-old male apoE^−/−^ mice (C57BL/6 genetic background) were purchased from Beijing HFK Bioscience Co., Ltd. (Beijing, China). All mice were housed in a specific pathogen-free environment at 18–23 °C under a 12 h light/dark cycle and allowed free access to normal chow ad libitum. After 1 week of adaption, apoE^−/−^ mice in the experimental group were fed with a high-fat diet (HFD), which consisted of a standard diet supplemented with 21% fat, 2% cholesterol and 5% lard oil, for 12 weeks to induce atherosclerosis. ApoE^−/−^ mice in control group were fed with a normal chow diet. At week 12, 16 ApoE^−/−^ mice 8 in HFD group and 8 in normal diet group were euthanized to measure miR-26a expression. Subsequently, the apoE^−/−^ mice with HFD were randomly divided into five groups (n = 8/group): control, miR-26a agomir negative control (AG-NC), miR-26a agomir (AG), miR-26a antagomir negative control (AN-NC) and miR-26a antagomir (AN) (all from Genepharma Co., Ltd., Shanghai, China). The sequence of miR-26a agomir was 5′-UUCAAGUAAUCCAGGAUAGGCU-3′. The sequence of miR-26a agomir negative control was 5′-UUCUCCGAACGUGUCACGUTT-3′. The sequence of miR-26a antagomir was 5′-AGCCUAUCCUGGAUUACUUGAA-3′. miR-26a antagomir negative control sequence was 5′-CAGUACUUUUGUGUAGUACAA-3′. ApoE^−/−^ mice received a tail vein injection with 10 mg/kg miR-26a agomir, miR-26a agomir negative control, miR-26a antagomir, or miR-26a antagomir negative control for 10 days. Control ApoE^−/−^ mice were administrated with equal amount of PBS at tail vein. At the end of the experiments, mice were killed for further analysis.

### Atherosclerotic lesion analysis

The animals were sacrificed by euthanasia and aortic sinuses were excised. Aortic sinuses were immediately fixed with 4% paraformaldehyde, embedded in an optimum cutting temperature (OCT) compound, and cut into 7 μm thick sections. To examine the atherosclerotic lesions of the aortic sinuses, sections of the aortic sinuses were stained with hematoxylin–eosin (HE) (Beyotime Institute of Biotechnology, Haimen, China) for 20 min and observed using an Olympus fluorescent microscope (Olympus Corp., Tokyo, Japan). To assess atherosclerotic plaque formation in atherosclerotic lesions, the lesion area in the aortic sinuse sections was dehydrated for 5 min in 100% isopropanol and measured following 0.5% Oil-Red-O (Sigma-Aldrich, St. Louis, MO, USA) staining using computer-assisted image quantification with Image Pro Plus 6.0 (Media Cybernetics, Inc., Rockville, MD, USA).

### Serum analysis

Mice were fasted for 8 h at the end of the 12 weeks study. Then blood samples were collected by retro-orbital venous plexus puncture after completion of treatment and the serum was separated by centrifugation. The serum concentrations of total cholesterol (TC), triglyceride (TG), low-density lipoprotein cholesterol (LDL-C), and high-density lipoprotein cholesterol (HDL-C) levels were measured using a colorimetric enzymatic assay system (Roche Modular P-800; Roche Diagnostics, Basel, Switzerland).

### Enzyme-linked immunosorbent assay (ELISA) analysis

The concentrations of secreted pro-inflammatory cytokines including tumor necrosis factor-α (TNF-α), interleukin-1β (IL-1β), IL-6, and chemokine monocyte chemoattractant protein 1 (MCP-1) in serum and supernatant of ECs were determined by commercially available ELISA kit (R&D Systems, Minneapolis, MN, USA).

### Cell culture and transfection

Human aortic endothelial cells (HAECs) were obtained from the American Type Culture Collection (Manassas, VA, USA) and cultured in RPMI-1640 medium (Thermo Fisher Scientific, Inc., Waltham, MA, USA) supplemented with 10% fetal bovine serum (FBS; Gibco, Carlsbad, CA, USA), and 1% penicillin/streptomycin (Invitrogen, CA, Carlsbad, USA) at 37 °C in a 5% CO_2_ atmosphere. HAECs were transfected with miR-26a mimics (miR-26a), miRNA negative control (miR-NC), miR-26a inhibitor (anti-miR-26a), inhibitor negative control (anti-miR-NC), pcDNA-TRPC3 (TRPC3), pcDNA empty control (Empty), siRNA against TRPC3 (si-TRPC3), siRNA negative control (si-NC) (all from Genepharma Co., Ltd.) using Lipofectamine 2000 (Invitrogen). At 48 h posttransfection, cells were treated with or without 50 nM ox-LDL (Beijing Xiesheng Bio-Technology Limited, Beijing, China) for further 24 h.

### Cell viability assay

MTT assay was conducted to examine cell viability of HAECs. Briefly, transfected cells were seeded in 96-well plates with a density of 3 × 10^4^ cells/well. At 24 h after ox-LDL treatment, a total of 20 μl of MTT solution (Sigma-Aldrich) was added to each well and incubated for 4 h at 37 °C. The supernatant was then removed and 150 μl of dimethyl sulfoxide (DMSO; Sigma-Aldrich) was added to dissolve the formazan products. Absorbance at 570 nm was measured using an ELx808 absorbance microplate reader (Bio-Tek Instruments, Inc., Winooski, VT, USA).

### Reverse-transcription quantitative polymerase chain reaction (RT-qPCR)

Total RNA was isolated from the aorta tissues and HAECs using Trizol (Invitrogen). To detect miR-26a expression, 10 ng of total RNA was immediately reverse-transcribed into cDNA using the reverse transcription kit (Takara, Dalian, China) and a specific stem-loop primer. The expression of miR-26a was then detected using SYBR^®^ Premix EX Taq™ II qPCR mix (Takara) on the GeneAmp^®^ PCR System 9700 (Applied Biosystems, Foster City, CA). PCR reaction were performed under the following conditions: 95 °C for 5 min, followed by 40 cycles of 95 °C for 10 s, 60 °C for 20 s and 72 °C for 20 s. The relative miR-26a expression level was calculated using 2^−△△Ct^ method and normalized against U6 small nuclear RNA (snRNA). The primers used were as below: miR-26a, forward 5′-GGA TCC GCA GAA ACT CCA GAG AGA AGG A-3′ and reverse 3′-AAG CTT GCC TTT AGC AGA AAG GAG GTT-5′; U6 primer, forward 5′-ATC CGC AAA GAC CTG T-3′; and reverse 5′-GGG TGT AAC ACT AAG-3′.

### Western blot analysis

Human aortic endothelial cells were lysed in the RIPA buffer (Pierce, Rockford, IL, USA) in the presence of a protease inhibitor cocktail (Pierce). Supernatants were collected by centrifugation at 12,000 rpm for 10 min at 4 °C and quantified using the Bradford assay (Bio-Rad). After denaturation, equal amount of proteins (20 μg) were separated by 10% sodium dodecyl sulfate–polyacrylamide gel electrophoresis (SDS–PAGE) and transferred onto a polyvinylidene difluoride membrane (PVDF; 0.22 μm, Millipore, Billerica, MA, USA). Following blocked with Tris-buffered saline containing Tween-20 and 5% skimmed milk at 4 °C overnight, the membranes were blotted with primary antibodies against TRPC3 (Abcam, Cambridge, MA, USA), phosphorylated p65 (p-p65), p65 (Abcam), phospholyrated IκBα (p-IκBα) and IκBα (Abcam). After washing with PBS buffer, the blots were incubated with horseradish peroxidase (HRP)-conjugated secondary antibody (Abcam). The protein signals were detected using chemiluminescent ECL reagent (Millipore).

### Terminal deoxynucleotidyl transferase dUTP nick end labeling (TUNEL) assay

TUNEL assay was performed to detect the apoptotic cells using the In Situ Cell Death Detection Kit (Roche Applied Science, Indianapolis, IN, USA). The treated HAECs grown on coverslips were fixed with 4% paraformaldehyde, followed by permeabilization with 0.1% Triton X-100. Then, cells were incubated with TUNEL reaction mixture at 37 °C for 1 h. The TUNEL-positive cells were counted in six to ten random selected fields under an Olympus fluorescent microscope (Olympus Corp.).

### Luciferase reporter assay

The wild-type or mutated 3′UTR sequences of TRPC3 containing the predicted miR-26a binding sites were cloned into the downstream of luciferase gene in the pmirGLO dual luciferase miRNA target expression vector (Promega, Madison, WI, USA) and named as WT-TRPC3 and MUT-TRPC3. For luciferase reporter assay, HAECs were seeded into a 96-well plate and cotransfected with 0.5 μg WT-TRPC3 or MUT-TRPC3 together with 20 mM miR-26a or miR-NC using Lipofectamine 2000 (Invitrogen). The cells were collected at 48 h posttransfection, and firefly and renilla luciferase activities were evaluated using Dual-Luciferase Reporter Assay System (Promega). Renilla luciferase activity was used as the normalization.

### Statistical analysis

All experimental results are presented as mean ± standard deviation (SD). Statistical analyses were carried out with two-tailed Student’s *t* test or one-way analysis of variance (ANOVA) using SPSS software (version 16; SPSS, Inc., Chicago, IL, USA). Differences were considered to be statistically significant at *P* < 0.05.

## Results

### miR-26a overexpression attenuated hyperlipidemia in HFD-treated apoE−/− mice

**Fig. 1:**
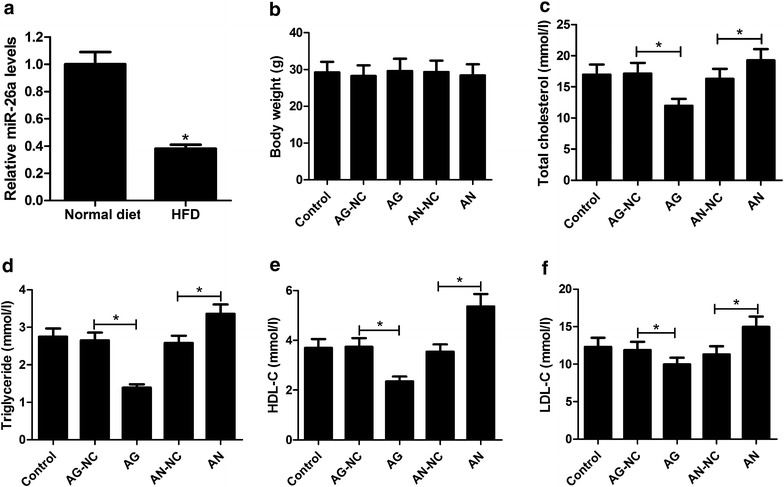
Effects of miR-26a on hyperlipidemia in HFD-treated apoE^−/−^ mice. **a** RT-qPCR analysis of miR-26a expression in apoE^−/−^ mice fed with HFD or normal diet. **b** The body weight of atherosclerosis mice in control, AG, AG-NC, AN, and AN-NC groups. The serum concentrations of TC (**c**), TG (**d**), HDL-C (**e**), and LDL-C (**f**) in atherosclerosis mice in control, AG, AG-NC, AN, and AN-NC groups. AG: miR-26a agomir, AG-NC: miR-26a agomir negative control, AN: miR-26a antagomir, AN-NC: miR-26a antagomir negative control, *P < 0.05

To determine the role of miR-26a in the pathogenesis of atherosclerosis, the apoE^−/−^ mice were fed with HFD to induce atherosclerosis and then tail vein injected with AG or AN. The expression of miR-26a was markedly reduced in atherosclerosis mice when compared to that in apoE^−/−^ mice fed with a normal diet (Fig. 1a), as demonstrated by RT-qPCR. Subsequently, gain-of-function and loss-of-function experiments were performed in atherosclerosis mice. However, the body weight of apoE^−/−^ mice in all groups including control, AG, AG-NC, AN, and AN-NC exhibited no obvious change (Fig. 1b). The effects of miR-26a on hyperlipidemia in apoE^−/−^ mouse model were further explored by measuring the serum lipid levels of clinical atherogenic factors including TC, TG, LDL-C, and HDL-C. ELISA results showed that injection with AG significantly decreased the concentrations of TC, TG, LDL-C, and HDL-C compared with AG-NC group, while AN treatment exerted the reverse effects on the concentrations of TC, TG, HDL-C, and LDL-C (Fig. 1c–f), suggesting that miR-26a overexpression inhibited lipid levels in apoE^−/−^ mouse model. Therefore, we concluded that miR-26a overexpression inhibited hyperlipidemia in HFD-treated apoE^−/−^ mice.

### miR-26a overexpression inhibited atherosclerotic lesion

**Fig. 2:**
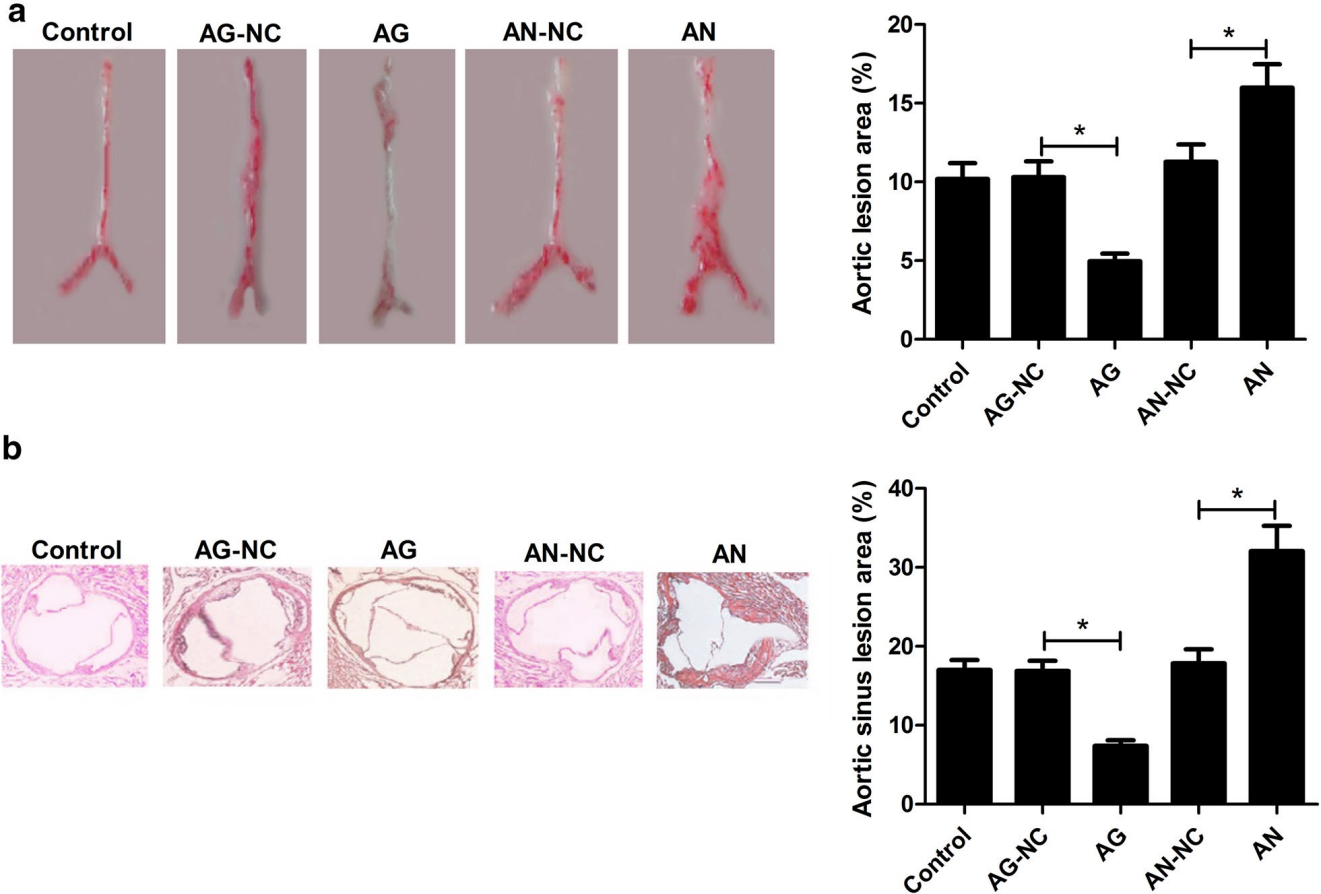
Effects of miR-26a on atherosclerotic lesion in ApoE^−*/*−^ mice. **a** Representative Oil-red-O staining of aorta in control, AG, AG-NC, AN, and AN-NC groups are shown. Atherosclerotic lesion area indicates the level of atherogenesis. **b** Characterization of aortic sinus atherosclerotic lesion area by HE staining in control, AG, AG-NC, AN, and AN-NC groups. *P < 0.05

We further examined the effects of miR-26a on atherosclerotic lesion by Oil-red-O and HE staining of aortic sinuses. To evaluate whether miR-26a could inhibit atherosclerotic plaque formation in aortas of apoE^−/−^ mice, we measured total atherosclerotic lesion area to evaluate the level of atherogenesis. As presented in Fig. 2a, a significant decrease of atherosclerotic plaque formation in thoracoabdominal aortas was observed in AG group relative to AG-NC group. On the contrary, AN-treated apoE^−/−^ mice showed an obvious increase of atherosclerotic plaque formation in comparison with AN-NC group. Moreover, HE staining found that atherosclerotic lesions in aortic roots were significantly lower in AG mice but higher in AN mice when compared with respective controls (Fig. 2b). Taken together, these results demonstrated that miR-26a overexpression inhibited atherosclerotic lesion.

### miR-26a overexpression suppressed inflammatory response in HFD-treated apoE^−/−^ mice

**Fig. 3:**
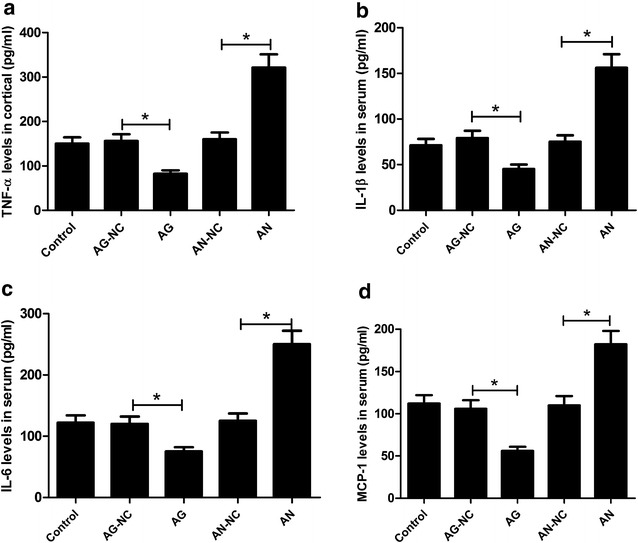
Effects of miR-26a on the production of inflammatory factors in HFD-treated apoE^−/−^ mice. ELISA assay of concentrations of TNF-α (**a**), IL-1β (**b**), IL-6 (**c**), and MCP-1 (**d**) in the plasma of HFD-treated apoE^−/−^ mice from control, AG, AG-NC, AN, and AN-NC groups. *P < 0.05

It is widely accepted that chronic lipid-induced inflammation plays a crucial role in the initiation and progression of atherosclerosis [18]. Thus, we analyzed the effect of miR-26a on inflammation in HFD-treated apoE^−/−^ mice. As compared with AG-NC group, AG treatment dramatically reduced the serum levels of inflammatory mediators including TNF-α (Fig. 3a), IL-1β (Fig. 3b), IL-6 (Fig. 3c), and MCP-1 (Fig. 3d) in HFD-treated apoE^−/−^ mice, as illustrated by ELISA analysis. However, the concentrations of TNF-α, IL-1β, IL-6, and MCP-1 in the plasma of HFD-treated apoE^−/−^ mice were strikingly improved by AN injection versus AN-NC group. All these data indicated that miR-26a overexpression suppressed inflammatory response in HFD-treated apoE^−/−^ mice.

### miR-26a overexpression attenuated the development of atherosclerosis in ox-LDL-treated HAECs

**Fig. 4:**
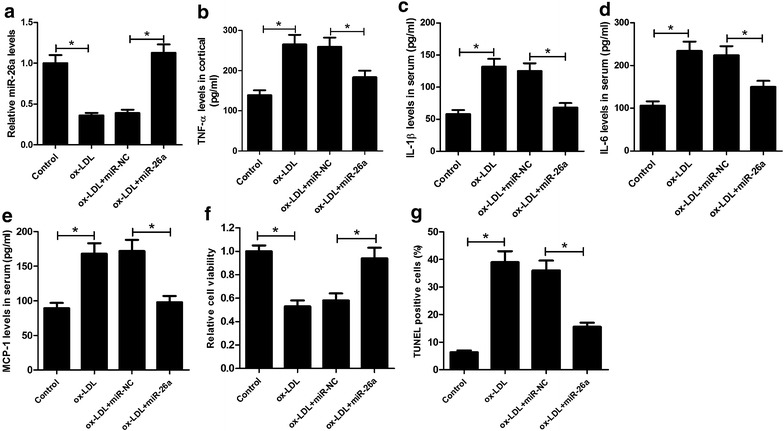
miR-26a overexpression attenuated the development of atherosclerosis in ox-LDL-treated HAECs. HAECs were transfected with miR-26a or miR-NC, followed by treatment with 50 nM ox-LDL for 24 h. **a** RT-qPCR analysis of miR-26a expression in treated HAECs. The concentrations of TNF-α (**b**), IL-1β (**c**), IL-6 (**d**), and MCP-1 (**e**) in the supernatant of treated HAECs were measured by ELISA assay. **f** Cell viability of treated HAECs was estimated by MTT assay. **g** Apoptosis of treated HAECs was assessed by TUNEL assay. *P < 0.05

To examine the effects of miR-26a on the progression of atherosclerosis in vitro, HAECs were transfected with miR-26a or miR-NC, followed by treatment with 50 nM ox-LDL, a key atherosclerotic risk factor that contributes to the pathogenesis and development of atherosclerosis [19], for 24 h. RT-qPCR results demonstrated that ox-LDL administration significantly reduced the expression of miR-26a in HAECs, while miR-26a transfection greatly restored the expression of miR-26a repressed by ox-LDL (Fig. 4a). In addition, ELISA results demonstrated that ox-LDL treatment markedly promoted the production of inflammatory factors including TNF-α (Fig. 4b), IL-1β (Fig. 4c), IL-6 (Fig. 4d), and MCP-1 (Fig. 4e) in the supernatant of HAECs, whereas transfection of miR-26a in HAECs prominently reversed ox-LDL-induced inflammatory response. Moreover, we further detected the effects of miR-26a on cell viability and apoptosis in ox-LDL-treated HAECs. As expected, MTT assay indicated that cell viability was notably impeded in ox-LDL treated HAECs in contrast to control cells, while the lowered cell viability was conspicuously recuperated following ectopic expression of miR-26a (Fig. 4f). Meanwhile, TUNEL assay suggested that ox-LDL treatment apparently induced apoptosis of HAECs when compared with control group, which was markedly attenuated by restoration of miR-26a expression (Fig. 4g). Collectively, these findings demonstrated that miR-26a overexpression attenuated the development of atherosclerosis in ox-LDL-treated HAECs.

### miR-26a suppressed TRPC3 expression in HAECs

**Fig. 5:**
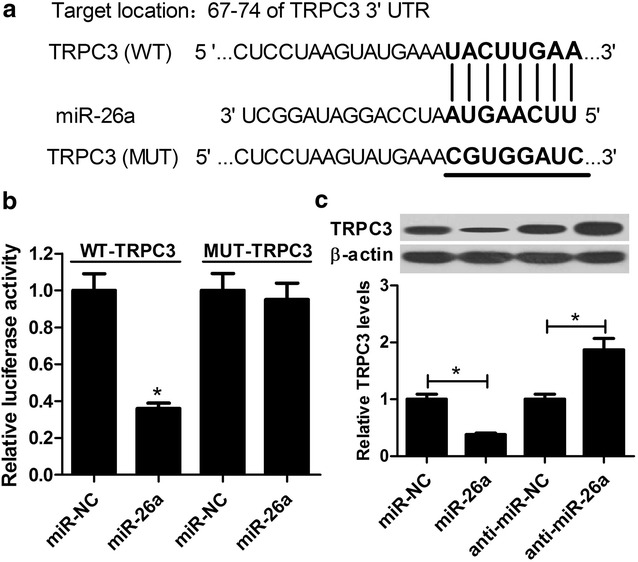
TRPC3 was a target of miR-26a in HAECs. **a** Schematic of the interaction sites of wild-type or mutated TRPC3-3′UTR with miR-26a. **b** Luciferase activity was measured by luciferase reporter assay in HAECs after cotransfection with WT-TRPC3 or MUT-TRPC3 and miR-26a or miR-NC. **c** The protein level of TRPC3 in HAECs treated with miR-26a, anti-miR-26a, or corresponding controls were determined by western blot. *P < 0.05

To elucidate the molecular mechanism by which miR-26a attenuated the development of atherosclerosis, we predicted the potential targets of miR-26a by miRanda (http://www.microrna.org/microrna/home.do) target-gene prediction software. According to the prediction results, TRPC3 was found to be a potential target of miR-26a (Fig. 5a). To confirm the direct binding between miR-26a and TRPC3, luciferase reporter plasmids containing the wild-type or mutated miR-26a binding sites in the 3′UTR of TRPC3 were constructed. HAECs were cotransfected with WT-TRPC3 or MUT-TRPC3 and miR-26a or miR-NC and luciferase reporter assay was performed. The results revealed that forced expression of miR-26a remarkably inhibited the luciferase activity of WT-TRPC3, but had no inhibitory effect on luciferase activity of MUT-TRPC3 (Fig. 5b). Next, we detected the protein levels of TRPC3 in HAECs transfected with miR-26a, anti-miR-26a, or matched controls. Western blot analysis proved that TRPC3 protein level was markedly decreased in miR-26a-transfected HAECs but significantly increased in anti-miR-26a-treated HAECs (Fig. 5c). Together, TRPC3 was a target of miR-26a in HAECs.

### miR-26a overexpression promoted cell viability and inhibited apoptosis in ox-LDL-treated HAECs by targeting TRPC3

**Fig. 6:**
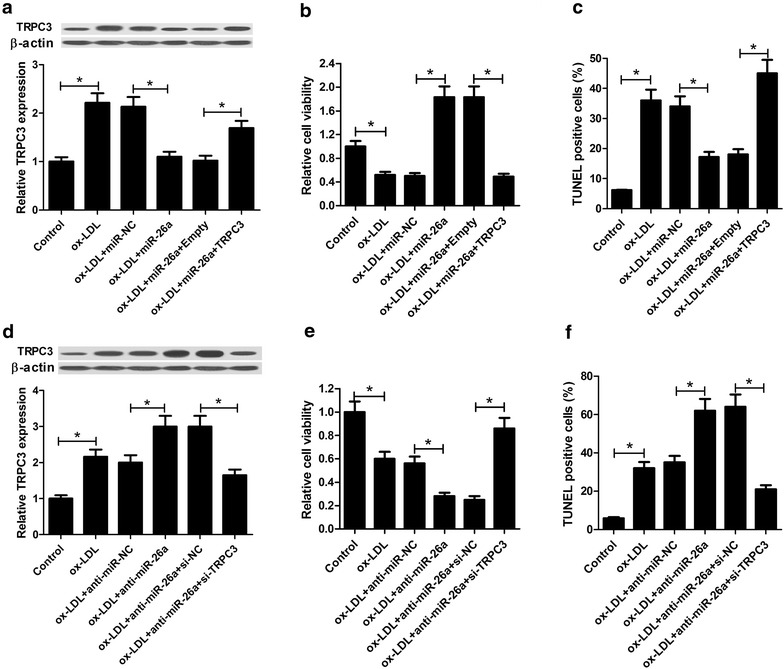
miR-26a overexpression promoted cell viability and inhibited apoptosis in ox-LDL-treated HAECs by targeting TRPC3. HAECs transfected with miR-26a, or together with TRPC3 were stimulated with ox-LDL for 24 h, followed by western blot analysis of TRPC3 protein level (**a**), MTT assay of cell viability (**b**), and TUNEL assay of apoptosis (**c**). HAECs introduced with anti-miR-26a, or combined with si-TRPC3 were treated with ox-LDL for 24 h, followed by western blot analysis of TRPC3 protein level (**d**), MTT assay of cell viability (E) and TUNEL assay of apoptosis (**f**). *P < 0.05

To address the role of TRPC3 in mediating the inhibitory effects of miR-26a on the development of atherosclerosis, HAECs were introduced with miR-26a, or together with TRPC3, as well as anti-miR-26a, or combined with si-TRPC3, and then stimulated with ox-LDL for 24 h. Western blot results demonstrated that the protein level of TRPC3 was significantly increased in HAECs after stimulation with ox-LDL. miR-26a overexpression markedly restrained the TRPC3 level in ox-LDL-treated HAECs, which was partially reverted by transfection with TRPC3-overexpressing plasmid (Fig. 6a). Conversely, miR-26a inhibition noticeably promoted TRPC expression in ox-LDL-treated HAECs, while TRPC3 knockdown remarkably reversed the effect of miR-26 inhibition on TRPC expression (Fig. 6d). As compared with miR-NC-transfected cells, increased expression of miR-26a promoted cell viability in ox-LDL-treated HAECs, which was significantly overturned by TRPC3 overexpression (Fig. 6b). On the contrary, miR-26a inhibition obviously suppressed cell viability in ox-LDL-treated HAECs compared to anti-miR-NC group, while the repressed cell viability was markedly recuperated by TRPC3 knockdown (Fig. 6e). TUNEL assay further uncovered that TRPC3 overexpression dramatically abrogated the inhibitory effect of miR-26a restoration on ox-LDL-induced apoptosis in HAECs (Fig. 6c), whereas TRPC3 knockdown weakened anti-miR-26a-induced apoptosis in ox-LDL-treated HAECs (Fig. 6f). These results suggested that miR-26a overexpression promoted cell viability and inhibited apoptosis in ox-LDL-treated HAECs by targeting TRPC3.

miR-26a overexpression inhibited activation of the nuclear factor-kappa B (NF-κB) pathway by downregulating TRPC3 in ox-LDL-treated HAECs.

**Fig. 7:**
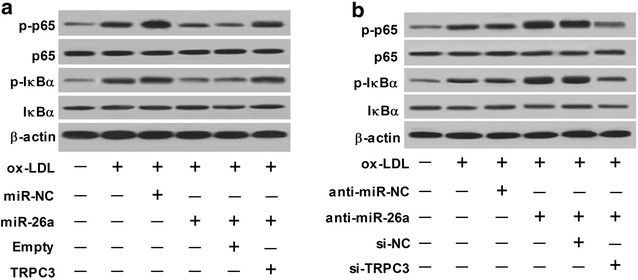
miR-26a overexpression inhibited activation of the nuclear factor-kappa B (NF-κB) pathway by downregulating TRPC3 in ox-LDL-treated HAECs. **a** HAECs were transfected with miR-26a, miR-NC, miR-26a + Empty, or miR-26a + TRPC3, followed by treatment with 50 nM ox-LDL for 24 h. Then, the protein levels of p65, p-p65, IκBα and p-IκBα in the treated HAECs were detected by western blot. **b** HAECs were transfected with anti-miR-26a, anti-miR-NC, anti-miR-26a + si-NC, or anti-miR-26a + si-TRPC3, followed by treatment with 50 nM ox-LDL for 24 h. Then, the protein levels of p65, p-p65, IκBα and p-IκBα in the treated HAECs were detected by western blot

Considering that NF-κB pathway has been implicated in the induction of inflammatory mediators and aberrant NF-κB activation can contribute to the initiation and progression of atherosclerosis [20], we thus hypothesized whether the activation of NF-κB pathway was involved in miR-26a-induced anti-inflammatory response in atherosclerosis. Western blot results showed that ectopic expression of miR-26a led to a significant reduction of the protein levels of p-p65 and p-IκBα but had no obvious effect on the protein levels of p65 and IκBα in ox-LDL-treated HAECs, suggesting that miR-26a overexpression significantly inhibited activation of the NF-κB pathway in atherosclerosis in vitro (Fig. 7a). However, TPRC3 overexpression remarkably abolished miR-26a-induced suppression of the NF-κB pathway. On the contrary, miR-26a suppression notably increased the protein levels of p-p65 and p-IκBα in ox-LDL-treated HAECs, which was markedly reversed by TRPC3 knockdown (Fig. 7b). Collectively, these results demonstrated that miR-26a overexpression inhibited activation of the NF-κB pathway by downregulating TRPC3 in ox-LDL-treated HAECs.

## Discussion

It is well-established that atherosclerosis is a complex arterial disease and the pathogenesis of atherosclerosis is always a critical topic. However, the mechanism involved in the pathogenesis of atherosclerosis is still not fully understood. Our study demonstrated that miR-26a was downregulated in the aorta tissues from HFD-fed apoE^−/−^ mice and ox-LDL-treated HAECs. miR-26a overexpression attenuated hyperlipidemia, decreased atherosclerotic lesion and inhibited inflammatory response in HFD-fed apoE^−/−^ mice. Also, miR-26a overexpression alleviated inflammatory response, improved cell viability and suppressed apoptosis in ox-LDL-treated HAECs. Moreover, the anti-arteriosclerotic effects of miR-26a were mediated partially through the regulation of its target TRPC3, providing the evidence again that miR-26a may be a potential therapeutic target for atherosclerosis.

ApoE is a 34-kDa secreted protein which is well defined to have anti-atherosclerotic activity. ApoE^−/−^ mice display high lipid levels, enhanced chronic inflammation, and atherosclerotic symptoms in response to spontaneous and diet-induced hypercholesterolemia [21], making it a widely applied in vivo model for investigating atherosclerosis [22]. Importantly, an initial step in the pathogenesis of atherosclerosis appears to be the injury of vascular endothelium [23], which might increase the risk of triggering atherosclerosis [24]. EC apoptosis plays a critical role in endothelial dysfunction and atherosclerosis lesion formation [25]. Ox-LDL is a key atherosclerotic risk factor that could induce EC apoptosis and promote the formation of foam cells [26]. Therefore, we used ox-LDL-stimulated HAECs as an in vitro cell model of atherosclerosis to simulate the pathological changes that occur in the early stage of atherosclerosis.

It has been well documented that aberrantly expressed miRNAs are closely associated with the pathogenesis and progression of atherosclerosis [27, 28]. miR-210 [29], miR-1185 [30], miR-19b [31], and let-7 g [32] have been reported to participate in the regulation of EC apoptosis in the setting of atherosclerosis. miR-19a [33], miR-155 [34] and miR-27 [35] were demonstrated to be able to modulate inflammatory reactions in atherosclerosis. In the present study, we demonstrated that miR-26a expression was lowered in the aorta tissues from HFD-fed apoE^−/−^ mice and ox-LDL-treated HAECs, the same with the previous study [14]. In vivo studies revealed that miR-26a overexpression attenuated hyperlipidemia by reducing the concentrations of TC, TG, LDL-C, and HDL-C, inhibited atherosclerotic lesion and inflammatory response in HFD-fed apoE^−/−^ mice, suggesting the anti-arteriosclerotic effects of miR-26a in vivo. Meanwhile, in vitro studies revealed that ectopic expression of miR-26a blocked ox-LDL-induced inflammatory response and apoptosis, relieved ox-LDL-elicited cell viability inhibition, and suppressed ox-LDL-induced activation of the NF-κB pathway in HAECs, illustrating the anti-arteriosclerotic effects of miR-26a in vitro. Previously, forced expression of miR-26a inhibited ox-LDL-induced EC apoptosis [14], consistent with the present study. Moreover, high level of miR-26a was reported to be associated with the endothelial protecting effect of tanshinol [36].

TRPCs have been proved to be involved in the pathogenesis of cardiovascular diseases, such as essential hypertension, cardiac hypertrophy, intima hyperplasia and endothelial dysfunction, and atherosclerosis [37]. TRPC3 has been documented to be implicated in atherosclerosis through regulating endothelium and macrophages [38]. A previous study demonstrated that TRPC3 had a dual beneficial effect on lesion progression by reducing cellularity at early stages and necrosis in the advanced plaques in apoe^−/−^ mouse model [17]. More interestingly, TRPC6 was previously identified as a target of miR-26a in ox-LDL-stimulated HAECs [14]. Therefore, we hypothesized whether miR-26a could also target TRPC3 to regulate the progression of atherosclerosis. As expected, our study confirmed that TRPC3 was a target of miR-26a and miR-26a repressed TRPC3 expression in HAECs, as demonstrated by luciferase reporter assay and western blot. Furthermore, TRPC3 overexpression markedly reversed the effects of miR-26a on cell viability and apoptosis in ox-LDL-stimulated HAECs, Moreover, TRPC3 overexpression reversed the inhibitory effect of miR-26a overexpression on the NF-κB pathway in ox-LDL-stimulated HAECs. These results suggested that miR-26a overexpression alleviated the pathogenesis of atherosclerosis by targeting TRPC3.

## Conclusions

In conclusion, our study demonstrated that miR-26a was downregulated in atherosclerosis and restoration of miR-26a expression alleviated the pathogenesis of atherosclerosis by targeting TRPC3, providing a new insight into the mechanism of miR-26a in the progression of atherosclerosis. Therefore, miR-26a may be a potential molecular target for the prevention and treatment of atherosclerosis.

## Abbreviations

TC: total cholesterol
TG: triglyceride
LDL-C: low-density lipoprotein cholesterol
HDL-C: high-density lipoprotein cholesterol
EC: endothelial cell
miRNAs: microRNAs
HFD: high-fat diet
TRPC6: transient receptor potential canonical 6
HAECs: human aortic endothelial cells
AN-NC: antagomir negative control
OCT: optimum cutting temperature
HE: hematoxylin–eosin
ELISA: enzyme-linked immunosorbent assay
